# Ameliorative Effect of Chrysin on Adenine-Induced Chronic Kidney Disease in Rats

**DOI:** 10.1371/journal.pone.0125285

**Published:** 2015-04-24

**Authors:** Badreldin H. Ali, Sirin A. Adham, Mohammed Al Za’abi, Mostafa I. Waly, Javed Yasin, Abderrahim Nemmar, Nicole Schupp

**Affiliations:** 1 Departments of Pharmacology and Clinical Pharmacy, College of Medicine and Health Sciences, Sultan Qaboos University, Muscat, Oman; 2 Department of Biology, College of Science, Sultan Qaboos University, Muscat, Oman; 3 Department of Food Science and Nutrition, College of Agricultural and Marine Sciences, Sultan Qaboos University, Muscat, Oman; 4 Departments of Internal Medicine, College of Medicine and Health Sciences, UAE University, Al Ain, United Arab Emirates; 5 Department of Physiology, College of Medicine and Health Sciences, UAE, Al Ain, United Arab Emirates; 6 Institute of Toxicology, University of Düsseldorf, Düsseldorf, Germany; The University of Manchester, UNITED KINGDOM

## Abstract

Chrysin (5, 7- dihydroxyflavone) is a flavonoid with several pharmacological properties that include antioxidant, anti-inflammatory and antiapoptotic activities. in this work, we investigated some effects of three graded oral doses of chrysin (10, 50 and 250 mg/kg) on kidney structure and function in rats with experimental chronic renal disease (CKD) induced by adenine (0.25% w/w in feed for 35 days), which is known to involve inflammation and oxidative stress. Using several indices in plasma, urine and kidney homogenates, adenine was found to impair kidney function as it lowered creatinine clearance and increased plasma concentrations of creatinine, urea, neutrophil gelatinase-associated lipocalin and *N*-Acetyl-beta-D-glucosaminidase activity. Furthermore, it raised plasma concentrations of the uremic toxin indoxyl sulfate, some inflammatory cytokines and urinary albumin concentration. Renal morphology was severely damaged and histopathological markers of inflammation and fibrosis were especially increased. In renal homogenates, antioxidant indices, including superoxide dismutase and catalase activities, total antioxidant capacity and reduced glutathione were all adversely affected. Most of these adenine – induced actions were moderately and dose -dependently mitigated by chrysin, especially at the highest dose. Chrysin did not cause any overt adverse effect on the treated rats. The results suggest that different doses of chrysin produce variable salutary effects against adenine-induced CKD in rats, and that, pending further pharmacological and toxicological studies, its usability as a possible ameliorative agent in human CKD should be considered.

## Introduction

Chronic kidney disease (CKD) is a major and growing public health problem [1–3] and is now considered a key determinant of the poor health outcomes of major non—communicable diseases [4]. The onset and progression of this disease, is affected by several factors, such as obesity, hypertension and diabetes mellitus [5–6]. The pathophysiological basis of the disease and its complication include inflammation and oxidative stress. These pathophysiological features are known to consistently occur in humans and animals [7–8]. They are also major mediators of the disease, exerting similar effects in a chronic renal failure (CRF) model in rats [9–11]. Patients and laboratory animals with CKD have high plasma concentrations of inflammatory mediators (such as C-reactive protein, tumor necrosis factor and other cytokines) and several markers of oxidative stress [12–13].

**Fig 1:**
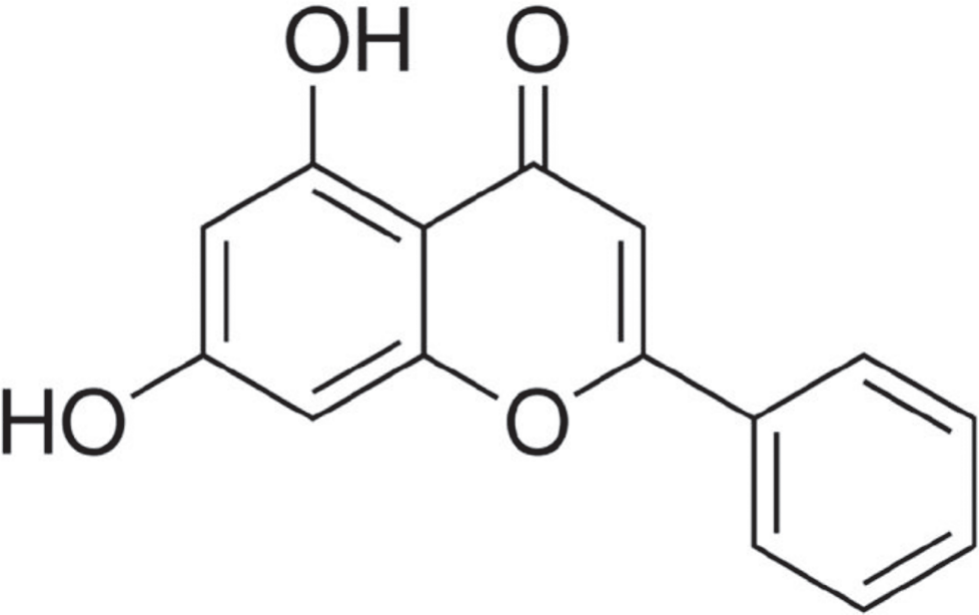
Chemical structure of chrysin (5,7 dihydroxy flavone).

Chrysin (5, 7-dihydroxyflavone) is an important natural plant flavonoid (Fig 1) with diverse pharmacological activities that include antioxidant [14], antiinflammatory [15], antiapoptotic [16], anti-atherogenic [17] and anti-cancer properties [18]. In rats, chrysin has also recently been shown to ameliorate cisplatin and doxorubicin nephrotoxicity [19] and methotrexate and carbon tetrachloride hepatotoxicity [20–21].

In the present work, and in continuation of our research program aiming at identifying novel agents for either the prevention or amelioration of adenine–induced CRD in a rat model [9,12,22–23], we have studied here the effects of treatment with this polyphenolic compound on rats with adenine–induced CRF, using several conventional and novel physiological, biochemical, immunological and histopathological methods, with a special focus on the possible effects of chrysin on the anti-inflammatory and antioxidant mechanisms in adenine – induced CKD.

## Materials and Methods

### Animals

Male Wistar rats (9–10 weeks old, initially weighting about 245 g) were obtained from the Animal Facility of Sultan Qaboos University (SQU), and housed in a room at a temperature of 22±2°C, relative humidity of about 60%, with a 12 h light–dark cycle (lights on at 6:00 am), with free access to standard pellet chow diet containing 0.85% phosphorus, 1.12% calcium, 0.35% magnesium, 25.3% crude protein and 2.5 IU/g vitamin D3 (Oman Flour Mills, Muscat, Oman) and tap water. Procedures involving animals and their care were conducted as described before [22] and an ethical approval from University Animal Research Ethics Committee was obtained (SQU/AEC/13/01).

### Experimental Design

Following an acclimatization period of one week, rats (n = 48) were randomly divided into eight equal groups and treated for five consecutive weeks. The 1^st^ group continued to receive the same diet without treatment until the end of the study (control group). The 2^nd^ group was switched to a powder diet containing adenine (0.25%^w/w^ in feed) for 35 days. The 3^rd^, 4^th^ and 5^th^ groups were given normal food and chrysin daily by gavage at doses of 10, 50 and 250 mg/kg, respectively. The 6^th^, 7^th^ and 8^th^ groups were given adenine in the feed as in the 2^nd^ group, plus chrysin at the above doses, respectively. The dose of adenine was chosen from our recent modified method for the induction of CKD [10], and the doses of chrysin were selected to bracket the doses reported in the literature [16].

The rats were weighed weekly during the experimental period. Rats were placed individually in metabolic cages for 24 h to collect urine. Thereafter, the rats were anesthetized with ketamine (75 mg/kg) and xylazine (5 mg/kg) both injected intraperitoneally, and blood (about 6 mL) was obtained from the anterior vena cava and placed into heparinized tubes. The blood and urine were spinned at 900 *g* at 4°C for 15 min. The plasma and the urine specimens were kept frozen at −80°C pending analysis within a month after the end of the experiment. The kidneys were removed, blotted on filter paper and weighed. A small piece of the right kidney was placed in formol-saline for subsequent histopathological examination. The rest of the kidneys were kept frozen at −80°C pending biochemical and molecular analysis and Western blot. The left kidney was homogenized in ice-cold Tris buffer (pH 7.4) to give a 10% w/v homogenate. The renal homogenates were centrifuged at 1500 *g* at 4°C for 15 min, and the supernatant obtained was used to measure several indices of the oxidant status.

### Biochemical Methods

The biochemical renal function tests in plasma and urine were measured using an automated analyzer as described before [22–23], except for plasma concentration of indoxyl sulfate (IS), which was assayed using an HPLC method, as previously described [24], and renal antioxidant and neutrophil gelatinase-associated lipocalin (NGAL), *N*-acetyl-β-D-glucosaminidase (NAG), and sclerostin, which were measured using ELISA.

### Histopathology

From the kidneys, 2 μm sections were cut and stained with hematoxylin and eosin (HE), periodic acid-Schiff stain (PAS) and Masson Trichome (MT) stain to assess inflmmatio, fibrosis, atrophy of basal membrane as well as dilatation as described earlier [9].

### Western blot analysis for caspase-3 and its cleaved isoform

Activation of the caspase cascade is a feature of apoptosis that is associated with CKD [10]. Therefore, we measured in this work the proteolytic activity of caspase-3 in the rat renal tissues collected from the eight different groups. The method used was described previously [22].

### Drugs and Chemicals

Adenine and chrysin were obtained from Sigma (St. Louis, MO, USA). All the ELISA enzymes and cytokines were obtained from Biovision (Mountain View, CA, USA), the except kits of sclerostin, NAG and NGAL, which were bought from Cloud –Clone Corp. (Houston, TX, USA), Diazyme (General Atomics, San Diego, CA, USA) and Bioporto diagnostics (Grusbakken, Gentofte, Denmark), respectively. Antioxidant kits were bought from Randox (Antrim, UK).

### Statistics

Data were expressed as means ± SEM, and were analyzed with GraphPad Prism Version 4.01 for Windows software (Graphpad Software Inc., San Diego, USA). Comparisons between groups were performed by analysis of variance (ANOVA), followed by Newman-Keuls multiple-range tests. *P* values <0.05 are considered significant.

## Results

### Physiological data

The general appearance of the adenine-treated rats was subjectively judged to be improved by chrysin treatment, especially at the highest dose (250 mg/kg). The kidneys from the control and chrysin-treated rats appeared normal. However, the kidneys of adenine-treated rats were pale, and a few crystals similar to those described for adenine were seen, mainly in the cortex area. The appearance of the kidneys of rats treated with adenine plus the three doses of chrysin were improved compared with the kidneys of rats treated with adenine alone.

The basic physiological data of the eight groups of rats in the experiment are shown in Table 1. Adenine treatment significantly reduced the growth of rats, and increased the absolute and relative kidney weight, the water intake and urine output (*P* < 0.05), but did not significantly affect either the feed intake or feces produced. Treatment with the three doses of chrysin caused a marked increase in the feed intake and body weights of rats, but did neither affect the water intake of rats, nor their urine output. However, simultaneous feeding of rats with adenine and chrysin mitigated the effects of adenine treatment, which were statistically different for the water and urine output when the highest dose of chrysin was used.

**Table 1:** Effect of chrysin treatment on some physiological parameters in rats with adenine-induced chronic kidney disease.

Group	Body weight change (%)	Relative kidney weight (%)	Water intake (mL)	Urine output (mL)
Control	28.5±2.3	0.7±0.02	21.8±2.3	9.6±1.2
Adenine	-5.1±1.9^a^	1.8±0.06^a^	55.2±0.96^a^	47.2±1.3^a^
Chrysin (10mg/Kg)	36.2±12.3^b^	0.6±0.01^b^	20.2±2.3^b^	8.5±0.1^b^
Chrysin (50mg/Kg)	38.6±10.3^b^	0.6±0.02^b^	22.0±1.8^b^	10.6±0.5^b^
Chrysin (250mg/Kg)	10.1±0.02^d^	0.5±0.01^b^	15.2±1.5^b^	7.4±0.6^b^
Chrysin (10mg/Kg)+Adenine	4.5±2.2^c^ ^d^	1.7±0.1^a^ ^c^ ^d^ ^e^	48.5±3.5^a^ ^c^ ^d^ ^e^	39.2±4.0^a^ ^c^ ^d^ ^e^
Chrysin (50mg/Kg)+Adenine	5.6±1.7^c^ ^d^	1.7±0.1^a^ ^d^ ^e^	45.0±4.1^a^ ^c^ ^d^ ^e^	38.2±2.6^a^ ^c^ ^d^ ^e^
Chryins (250mg/Kg)+Adenine	1.4±0.1^a^ ^c^ ^d^	1.3±0.5	35.6±5.5^c^ ^e^	26.3±3.3^a^ ^c^ ^d^ ^e^ ^f^ ^g^

Values in the tables are mean ± SEM (n = 6).

Chronic kidney disease (CKD) was induced by the inclusion of adenine in the feed at a concentration of 0.25% w/w for 35 days, and chrysin (10, 50, and 250mg/kg) was given orally by gavage either alone or concomitantly with adenine. On the last day of the treatment, the rats were placed in metabolic cages to collect urine.

Values with superscripts are statistically different *p* value

^a^ vs control

^b^ vs. Adenine

^c^ vs. Chrysin (10 mg/kg)

^d^ vs. Chrysin (50 mg/kg)

^e^ vs. Chrysin (250 mg/kg)

^f^ vs. Chrysin (10 mg/kg) + Adenine

^g^ vs. Chrysin (50 mg/kg) + Adenine.

### Biochemical measurements

**Fig 2:**
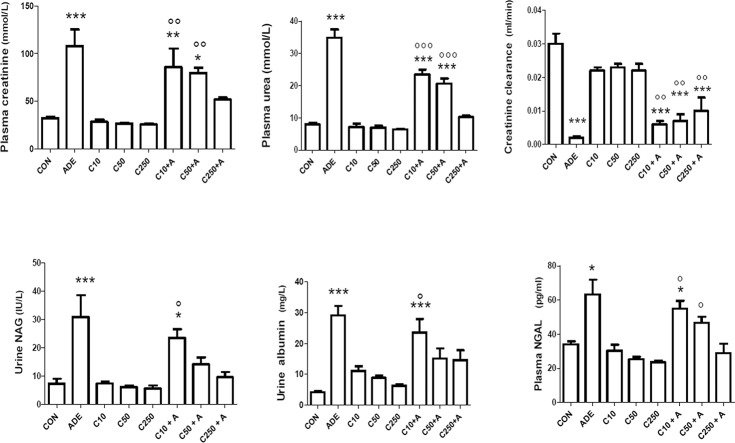
Effect of chrysin treatment on creatinine clearance, plasma concentrations of creatinine, urea and neutrophil gelatinase-associated lipocalin (NGAL), urinary albumin concentration and N-acetyl-β-D-glucosaminidase (NAG) activity in control rats and rats treated either singly or concomitantly with either adenine (ADE) or chrysin (C) at doses of 10, 50 or 250 mg/kg. Each column and vertical bar is a mean ± SEM (n = 6).

As shown in Fig 2, adenine treatment significantly increased creatinine and urea concentrations in plasma, and decreased creatinine clearance (*P* < 0.05). Adenine also increased the activities of urinary NAG and plasma NGAL. Treatment with the three doses of chrysin alone did not significantly affect any of the above indices, which were not significantly different from those of the controls (*P*> 0.1). Co-administration of chrysin at the three graded doses (10, 50 and 250 mg/kg) with adenine caused a significant and dose-dependent amelioration of all indices measured, except IS, which was insignificantly mitigated when compared with that in rats treated with adenine alone.

**Fig 3:**
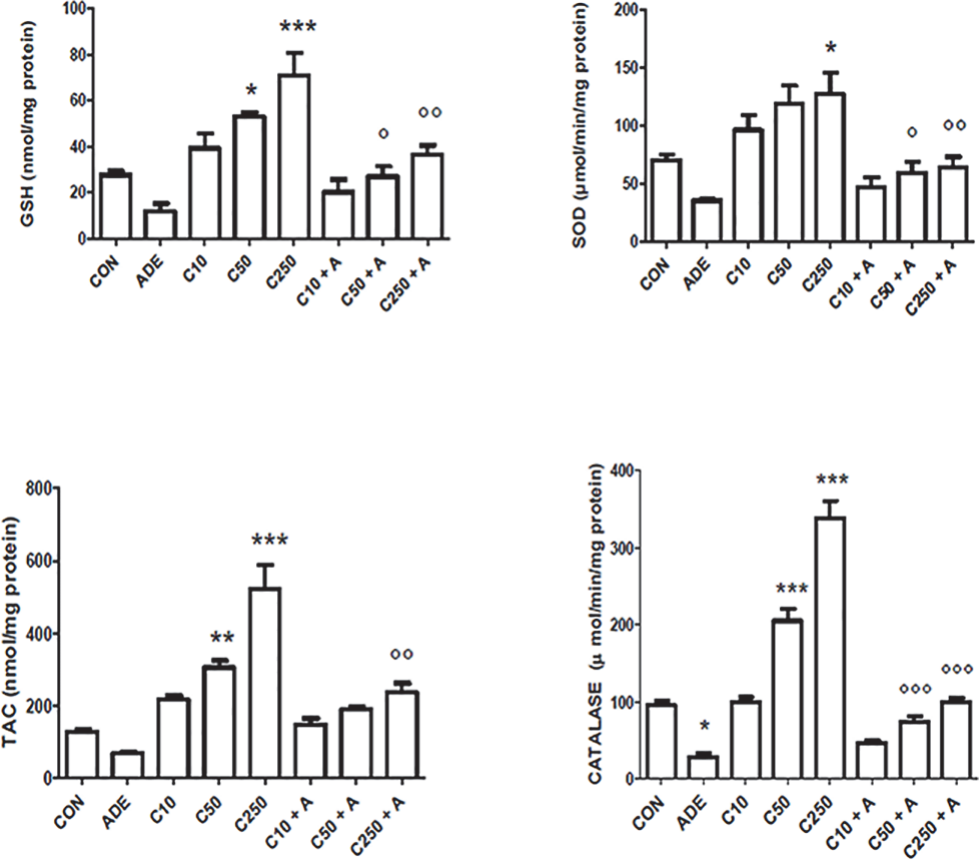
Effect of chrysin treatment on either renal concentration or activity of reduced glutathione (GSH), superoxide dismutase (SOD), total antioxidant capacity (TAC) and catalase (CAT) in control rats and rats treated either singly or concomitantly with either adenine (ADE) or chrysin (C) at doses of 10, 50 or 250 mg/kg. Each column and vertical bar is a mean ± SEM (n = 6).

The effect of chrysin treatment on antioxidant indices in kidney homogenates from control rats and rats with adenine-induced CKD is shown in Fig 3. Adenine treatment significantly depressed SOD and catalase activities, GSH concentrations, and total antioxidant capacity. Treatment with chrysin alone at the three graded doses significantly enhanced these indices in a dose-dependent manner. Concomitant treatment of rats with adenine and the three doses of chrysin dose-dependently abated the adenine-induced oxidative stress (*P*<0.05).

Table 2 shows the effect of the three doses of chrysin (with and without adenine co-treatment) on the activities of some enzymes in plasma. Adenine treatment caused significant and marked increases (about 2–4-fold) in the enzymes measured (ALT, AST, CK, GGT and LDH). The three doses of chrysin alone exerted no significant effect on any of the enzymatic activities. In the co-treated groups, the three doses of chrysin were effective in significantly ameliorating the action of adenine on the measured enzymes.

**Table 2:** Effect of chrysin on the activities of some enzymes in plasma rats with adenine-induced chronic kidney disease.

Enzyme	ALT (IU/L)	AST (IU/L)	CK (IU/L)	GGT (IU/L)	LDH (IU/L
Control	28.0±3.9	40.2±3.9	265.0±24.3	1.5±0.3	189.0±14.2
Adenine	113.2±24.9^a^	126.3±21.6^a^	453.3±13.5^a^	5.3±0.6^a^	358.7±16.9^a^
Chrysin (10mg/Kg)	53.2±4.7^b^	89.0±8.6	259.2±10.7^b^	1.7±0.3^b^	189.2±26.0^b^
Chrysin (50mg/Kg)	39.0±5.4^b^	86.7±7.5	262.5±20.9^b^	1.5±0.3^b^	174.4±13.4^b^
Chrysin (250mg/Kg)	33.3±0.7^b^	82.3±27.0	247.2±27.7^b^	1.2±0.5^b^	156.8±18.9^b^
Chrysin (10mg/Kg)+Adenine	71.8±7.8	102.0±1.8^a^	442.7±23.8^a^ ^c^ ^d^ ^e^	3.8±0.6^a^ ^d^ ^e^	320.0±43.1^a^ ^c^ ^d^ ^e^
Chrysin (50mg/Kg)+Adenine	50.0±5.2^b^	93.8±4.8	322.2±29.7^b^ ^f^	3.2±0.7	275.3±17.9^e^
Chryins (250mg/Kg)+Adenine	41.3±1.3^b^	66.3±3.2^b^	310.2±25.7^b^ ^f^	2.5±0.2^b^	221.6±29.3^b^

Values in the tables are mean ± SEM (n = 6).

Chronic kidney disease (CKD) was induced by the inclusion of adenine in the feed at a concentration of 0.25% w/w for 35 days, and chrysin (10, 50, and 250mg/kg) was given orally by gavage either alone or concomitantly with adenine. On the last day of the treatment, rats were killed for blood collection.

AST: aspartate aminotransferase, ALT: alanine aminotransferase, CK: creatinine kinase, GGT: gamma glutamyl transferase, LDH: lactate dehydrogenase.

Values with superscripts are statistically different *p* value

^a^ vs control

^b^ vs. Adenine

^c^ vs. Chrysin (10 mg/kg)

^d^ vs. Chrysin (50 mg/kg)

^e^ vs. Chrysin (250 mg/kg)

^f^ vs. Chrysin (10 mg/kg) + Adenine.

**Fig 4:**
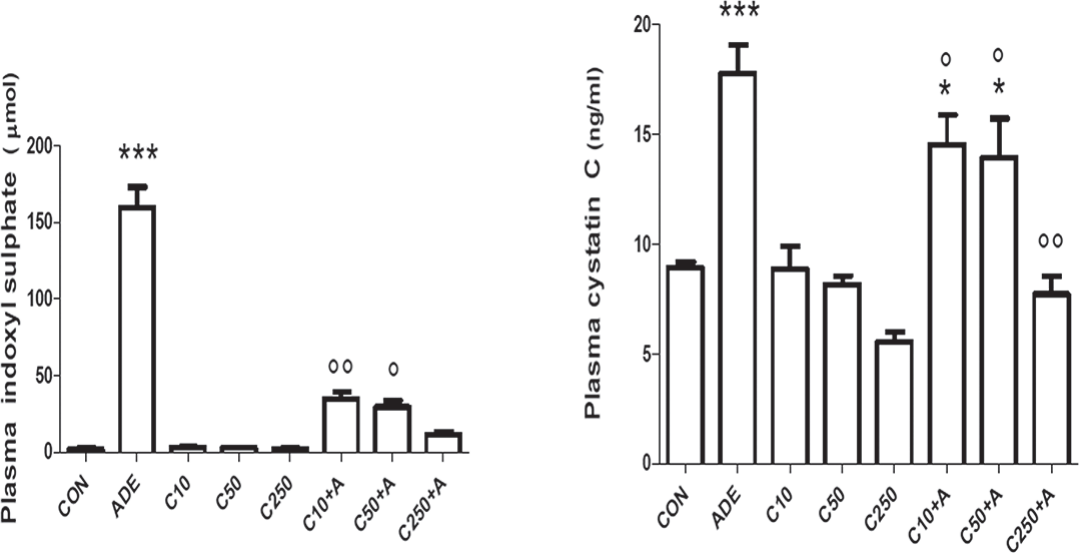
Effect of chrysin treatment on plasma concentrations of indoxyl sulfate and cystatin in control rats and rats treated either singly or concomitantly with either adenine (ADE) or chrysin (C) at doses of 10, 50 or 250 mg/kg. Each column and vertical bar is a mean ± SEM (n = 6).

Adenine treatment caused a significant increase in the concentrations of indoxyl sulfate (IS) and cystatin C in plasma (Fig 4). Chrysin alone did not significantly affect either the IS or cystatin C concentrations. However, when adenine was given together with chrysin, there was a dose-dependent diminution in its concentration, which was statistically significant at all doses of chrysin (in case of IS, (*P* < 0.001), and at the highest dose of chrysin (in case of cystatin C, *P* < 0.001).

**Fig 5:**
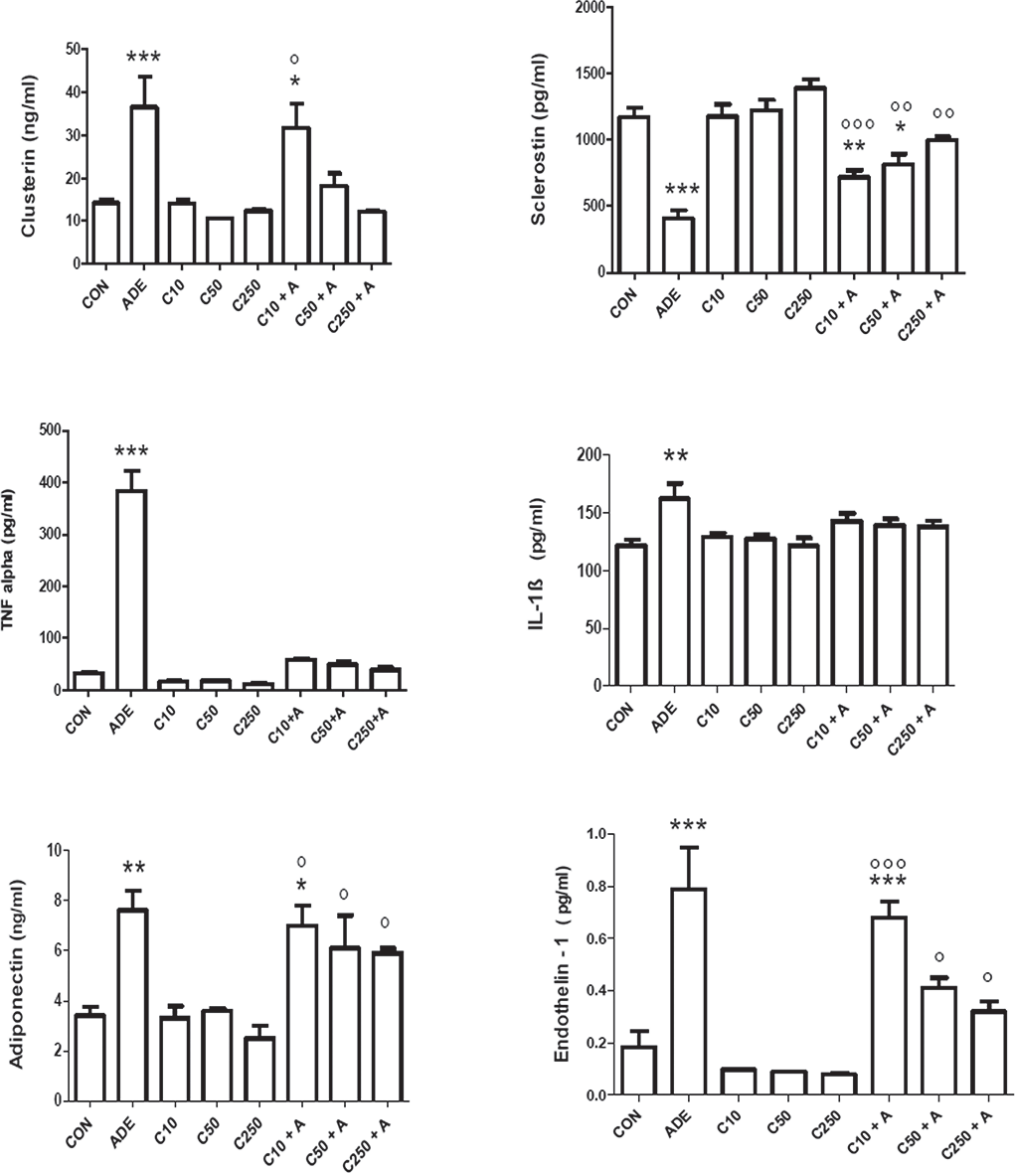
Effect of chrysin treatment on plasma concentrations of the cytokines tumor necrosis factor alpha (TNF α), sclerostin adiponectin, interleukin–one beta (IL-1β) and endothelin in control rats and rats treated either singly or concomitantly with either adenine (ADE) or chrysin (C) at doses of 10, 50 or 250 mg/kg. Each column and vertical bar is a mean ± SEM (n = 6).

As shown in Fig 5, adenine treatment significantly increased the plasma concentrations of endothelin – 1, adiponectin, TNF- α and IL—1β, and decreased that of sclerostin (*P* < 0.001). Treatment of rats with any of the three doses of chrysin alone did not significantly affect any of the above. Co-administration of chrysin and adenine produced a dose – dependent decrease in clusterin levels (*P* < 0.05) when compared with control rats and rats treated with chrysin alone. It did not significantly affect IL—1β concentrations, but it markedly and significantly decreased the TNF- α levels (*P* < 0.001) when compared with those in adenine – treated rats. The effect of chrysin treatment in the latter animals was less marked, and was only significant in the case of endothelin – 1, and at the highest dose of chrysin. Adenine significantly decreased the concentration of sclerostin, and co- treatment with chrysin antagonized this action in a dose –dependent fashion, which was statistically significant at doses of 50 and 250 mg/kg.

### Molecular analysis of apoptosis

**Fig 6:**
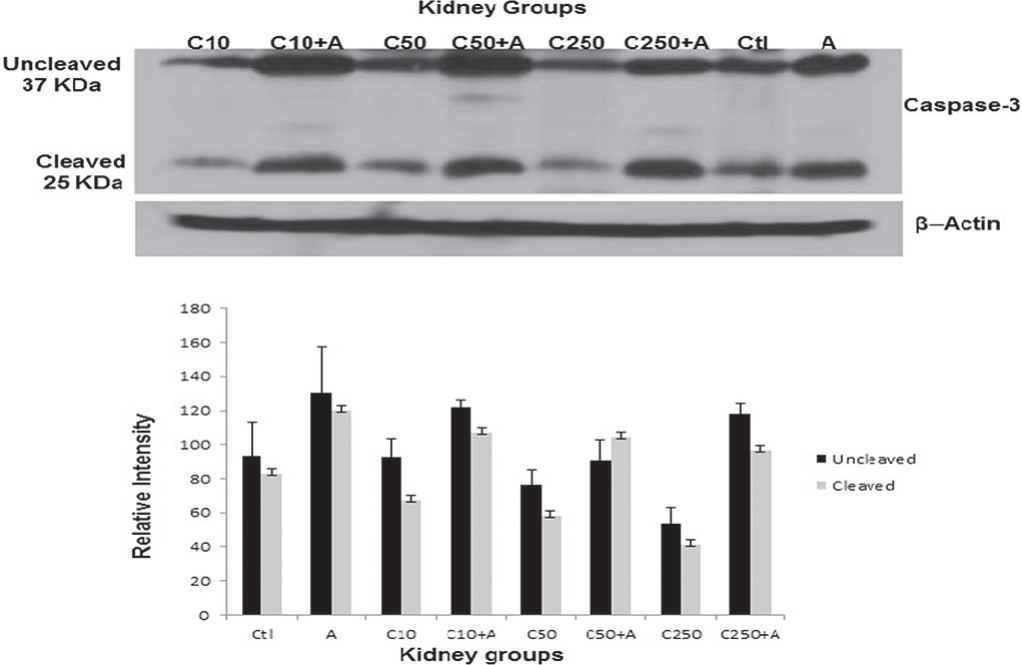
The blot key from left to right in panel A shows both the un-cleaved (37KDa) and cleaved (25KDa) caspase-3 bands in kidney homogenates from rats after their treatment with chrysin 10 mg/kg, chrysin 10 mg/kg + adenine, chrysin 50 mg/kg, chrysin 50 mg/kg + adenine, chrysin 250 mg/kg, chrysin 250 mg/kg + adenine, saline (control), and adenine using Western blot analysis. The graph in panel B represents the densitometry measurement of both the un-cleaved and cleaved caspase-3 bands in kidney homogenates from all treated and non-treated rats.

The results of this part are shown in Fig 6 and the graph (showing the mean relative intensity of the un-cleaved and cleaved bands of the caspase-3 protein as measured optically using Image J) indicates that treatment with chrysin alone at the three different doses used did not affect apoptosis significantly (*P* > 0.05) in rat kidneys as shown by the low intensity of the cleaved bands, which were comparable with control kidneys. However, the concomitant treatment with adenine and chrysin did not exhibit any significant improvement in the degree of apoptosis induced by adenine, which was represented by the thick cleaved bands of caspase-3 (25 KDa) that was comparable with the cleaved band obtained by the single treatment with 0.25% of adenine (far right of the blot).

### Histopathology

**Fig 7:**
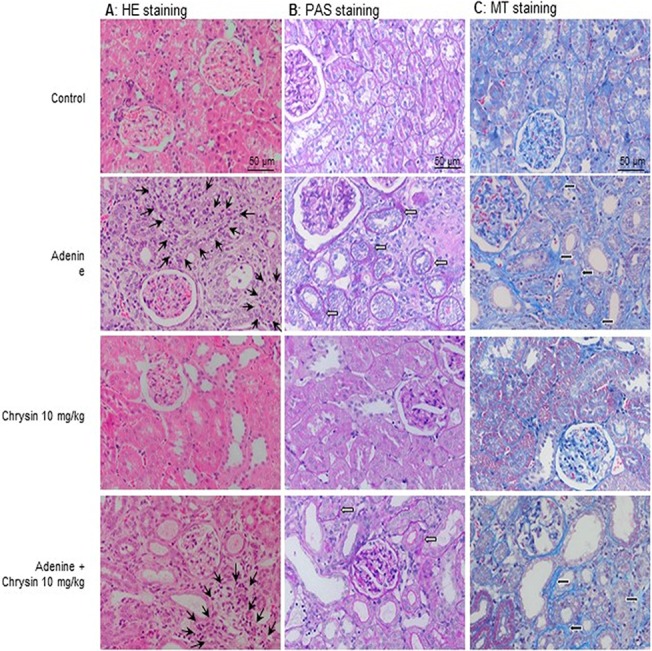
Effect of chrysin on adenine-induced morphological changes in the kidney. Representative pictures of kidney slices of the control group, the adenine group, the chrysin 10mg/kg group and the adenine plus chrysin 10 mg/kg group used for semi-quantitative scoring of inflammation and fibrosis. (A) HE staining used for the identification and semi-quantitative scoring of inflammation. The black slender arrows point to examples of leucocyte infiltration (20-fold magnification). (B) PAS staining, used for the identification and semi-quantitative scoring of atrophy of the basal membrane and dilatations. The white filled arrows point to examples of atrophic basal membranes (20-fold magnification). (C) MT staining, used for identification and semi-quantitative scoring of fibrosis. The black filled arrows point to collagen deposition, characteristic for fibrosis (20-fold magnification).

Histopathological examination of kidney sections from control rats showed no sign of damage. As shown in Table 3 and Fig 7, he kidneys of adenine-treated animals showed several signs of extensive damage, including inflammation, as well as fibrosis. Chrysin alone did not significantly alter the morphological appearance of the kidneys. In combination with adenine, the pathology was slightly ameliorated; the best was at the lowest concentration of chrysin.

**Table 3:** Effect pf chrysin on kidney morphology in rats with adenine-induced kidney chronic disease.

Group	Inflammation	Fibrosis	Atrophy	Dilatation
Control	0.08±0.02	0	0	0
Adenine	2.52±0.25^a^	3.03±0.18^a^	2.73±0.40^a^	0.20±0.05
Chrysin (10mg/Kg)	0.24±0.05	0	0.17±0.03	0
Chrysin (50mg/Kg)	0.24±0.06	0	0.23±0.07	0
Chrysin (250mg/Kg)	0.18±0.06	0.04±0.02	0.30±0.10	0
Chrysin (10mg/Kg)+Adenine	2.1±0.16^a^ ^c^	1.78±0.13^a^ ^b^ ^c^	1.7±0.21^a^ ^b^ ^c^	0.34±0.05^a^ ^c^
Chrysin (50mg/Kg)+Adenine	2.68±0.20^a^ ^d^ ^f^	2.35±0.12^a^ ^b^ ^d^ ^f^	2.31±0.17^a^ ^d^	0.59±0.05^a^ ^b^ ^d^ ^f^
Chryins (250mg/Kg)+Adenine	2.66±0.10^a^ ^e^ ^f^	2.67±0.10^a^ ^e^ ^f^	1.65±0.21^a^ ^b^ ^e^	0.54±0.07^a^ ^b^ ^e^ ^f^

Values in the tables are mean ± SEM (n = 6)

Chronic kidney disease was induced by inclusion of adenine in the feed at a concentration of 0.25 0% w/w for 35 days, and chrysin (10, 50 & 250 mg/kg) was given orally by gavage either alone or concomitantly with adenine. Histopathology was evaluated from kidney slices stained with hematoxylin, Masson trichrome and periodic schiff acid.

Values with superscripts are statistically different *p* value

^a^ vs control

^b^ vs. Adenine

^c^ vs. Chrysin (10 mg/kg)

^d^ vs. Chrysin (50 mg/kg)

^e^ vs. Chrysin (250 mg/kg)

^f^ vs. Chrysin (10 mg/kg) + Adenine

^g^ vs. Chrysin (50 mg/kg) + Adenine.

## Discussion

Nephroprotection using natural products (such as the flavonoid chrysin that we have investigated here) may be a safe, efficacious, and cost–effective option to protect kidneys against aggressive factors, and to obviate the progression of renal impairment to the stage of renal failure where either dialysis or kidney transplantation are the only available options. Both options may be costly or even unavailable in many parts of the developing countries [25–28].

This is, as far as we are aware, the first study on the effect of chrysin on an adenine-induced model for CRF. While this work was being written up, a paper on the beneficial effect of chrysin in experimental diabetic nephropathy was published [29], and was broadly in line with a few of the results we obtained here, but different with some.

In this work, rats were treated with adenine to induce CKD, and concomitantly give chrysin as a preventive agent. In a subsequent experiment we will give chrysin after the end of the adenine treatment to test its possible therapeutic action on CKD.

Adenine induced various pathological signs of CKD in the rats, as shown in previously published work [12,22]. In this work, we measured, for the first time in rats with adenine–induced CKD, the cytokine sclerostin, which is a newly–characterized blood marker of CKD in humans [30–31] and rats with 5/6-nephrectomy–induced CKD [32].

The modest beneficial effects seen in the present work are somewhat different from the results of Ahad et al [29], who reported that chrysin (at a single oral dose of 40 mg/kg for 16 consecutive weeks) completely prevented the development of diabetic nephropathy in rats. The possible reasons for the discrepancy between our results and those of Ahad et al, [29], are that diabetes nephropathy is not as damaging to the kidneys as adenine, and its nephropathy may be brought about by different mechanisms [33]. In addition, our treatment with the three graded doses was for five weeks. However, and in agreement with Ahad et al, [29], our results also indicated that adenine treatment has induced a highly significant increase in the concentrations of some inflammatory cytokines (particularly TNF–α), and this effect has been significantly and markedly suppressed by chrysin treatment at the three doses given. TNF–α is an important pro-inflammatory cytokine with prominent effects in many conditions [30], which include adenine – induced CKD [9]. The inhibitory effect of chrysin on TNF–α suggests that this phytochemical has exerted its ameliorative effect on adenine – induced CKD, at least partly, by an antiinflammatory action. However, chrysin in our work did not show a significant effect on the adenine –induced increase in the plasma concentration of the proinflammatory cytokine. The reason for this inconsistency with the results compared to the other proinflammatory cytokine TNF–α is not known. Recently the antiinflammatory action of chrysin has been confirmed in a mouse model of focal cerebral ischemia/ reperfusion injury by inhibiting the *NF –kB* signaling pathway, which is a regulator of the expression of *iNOS* and *COX-2*. [34]. In this work, we measured for the first time in rats with adenine–induced CKD the cytokine sclerostin, which is a newly–characterized blood marker of CKD in humans [30–31] and rats with 5/6-nephrectomy–induced CKD [32]. Our results confirm the usefulness of sclerostin as a biomarker for CKD. Although the full significance of its measurement in either plasma or serum is not known with certainty, it has been found to be associated with several biological and clinical data in hemodialysis patients [31,35]. Here, we found that adenine has depressed the level of sclerostin, and that co-administration of chrysin in adenine–treated rats also antagonized the adenine–induced suppression of sclerostin. It has been shown in a single report that the sclerostin plasma level is significantly depressed in rats with CKD induced surgically by nephrectomy [36], an action related to its role as A Wnt/β – catenin pathway inhibitor.

As has been found in our previous papers adenine induced a highly significant increase in the plasma concentration of the tryptophan – derived uremic toxin IS [9–10]. Chrysin significantly and dose- dependently reversed this action (Fig 4). The mechanism by which this reduction has occurred has not been investigated here, but it has been shown previously that the levels of IS are lowered by preventing the bacterial generation of indole or by absorbing the latter within the intestine [37–38].

The present results also suggest that chrysin had no adverse effect on the hepatic and renal functions of treated rats, nor on either their water and feed intake, or fecal and urinary output. The relative safety of this flavonoid suggests that, pending further pharmacological and toxicological investigations, it could be a candidate for further studies as a nephroprotectant agent in humans. It should be mentioned, however, that a recent *in vitro* study reported that, at a relatively high concentration, chrysin showed an inhibitory action on 6–phosphogluconate dehydrogenase activity in human erythrocytes [39]. The significance of this is not known, and warrants further studies *in vivo*.

In this work, chrysin was not able to ameliorate significantly the morphological damage induced by adenine in the kidney as well as the physiological and biochemical endpoints. Using molecular quantification of apoptosis, chrysin did not cause any significant increase in caspase-3 levels indicating, in part, its lack of toxicity to the kidneys, and this was also confirmed by light histopathology examination. On the other hand, it did not show any significant improvement in kidneys of rats treated with adenine. This result contradicts the observations of Ahad et al. [29], who noticed a marked improvement in histopathological changes in kidneys of diabetic rats. It has to be stated that from the appearance of the kidneys in Ahad´s study the damage inflicted to them by the diabetes was not as severe as the damage we observed in the adenine animals. So the relative amelioration might be similar. Further, we concentrated our examination on the tubular system, which is mainly affected by adenine, while Ahad et al. [29] showed mostly changes in glomeruli. An explanation for better renal function endpoints despite only slightly better histological appearance might be that histological lesions appear before the appearance of either proteinuria or renal functional deterioration, as extensively studied in renal transplant patients [40]. Thus, the kidney is able to compensate for its histological damage, and with chrysin, maybe this compensation was improved.

In conclusion, this work has shown that the three graded doses of chrysin were modestly effective in mitigating some of the actions of adenine–induced CKD in rats. Further studies are warranted to confirm the usefulness and safety of chrysin as either an adjunct agent for the therapy or prevention of this condition. Before extrapolating these results to humans with CKD, more research is needed to verify the safety of increasing doses of chrysin in volunteers. A single report studied the disposition and metabolism of chrysin in seven healthy volunteers, each receiving a single dose of about 5 mg/kg [41]. It was found that chrysin undergoes extensive metabolism, and has low bioavailability.

The molecular mechanism(s) of antiinflammatory and antioxidant actions of chrysin in adenine –induced CKD are not known with certainty and has not been studied here. However, several possible mechanisms have been suggested. These include modulating the peroxisome proliferator activated receptor gamma and decreasing the expressions of the pro-inflammatory nuclear factor kappa-B (NF-κB) signaling pathway [42–43].
